# MicroRNA Signature Characterizes Primary Tumors That Metastasize in an Esophageal Adenocarcinoma Rat Model

**DOI:** 10.1371/journal.pone.0122375

**Published:** 2015-03-31

**Authors:** Ali H. Zaidi, Lindsey T. Saldin, Lori A. Kelly, Linda Bergal, Ricardo Londono, Juliann E. Kosovec, Yoshihiro Komatsu, Pashtoon M. Kasi, Amit A. Shetty, Timothy J. Keane, Shyam J. Thakkar, Luai Huleihel, Rodney J. Landreneau, Stephen F. Badylak, Blair A. Jobe

**Affiliations:** 1 Esophageal and Lung Institute, Allegheny Health Network, Pittsburgh, Pennsylvania, United States of America; 2 McGowan Institute for Regenerative Medicine, University of Pittsburgh, Pittsburgh, Pennsylvania, United States of America; 3 Department of Medicine, University of Pittsburgh, Pittsburgh, Pennsylvania, United States of America; 4 Division of Gastroenterology, Allegheny Health Network, Pittsburgh, Pennsylvania, United States of America

## Abstract

**Objective:**

To establish a miRNA signature for metastasis in an animal model of esophageal adenocarcinoma (EAC).

**Background:**

The incidence of esophageal adenocarcinoma (EAC) has dramatically increased and esophageal cancer is now the sixth leading cause of cancer deaths worldwide. Mortality rates remain high among patients with advanced stage disease and esophagectomy is associated with high complication rates. Hence, early identification of potentially metastatic disease would better guide treatment strategies.

**Methods:**

The modified Levrat’s surgery was performed to induce EAC in Sprague-Dawley rats. Primary EAC and distant metastatic sites were confirmed via histology and immunofluorescence. miRNA profiling was performed on primary tumors with or without metastasis. A unique subset of miRNAs expressed in primary tumors and metastases was identified with Ingenuity Pathway Analysis (IPA) along with upstream and downstream targets. miRNA-linked gene expression analysis was performed on a secondary cohort of metastasis positive (n=5) and metastasis negative (n=28) primary tumors.

**Results:**

The epithelial origin of distant metastasis was established by IF using villin (VIL1) and mucin 5AC (MUC5AC) antibodies. miRNome analysis identified four down-regulated miRNAs in metastasis positive primary tumors compared to metastasis negative tumors: miR-92a-3p (p=0.0001), miR-141-3p (p=0.0022), miR-451-1a (p=0.0181) and miR133a-3p (p=0.0304). Six target genes identified in the top scoring networks by IPA were validated as significantly, differentially expressed in metastasis positive primary tumors: Ago2, Akt1, Kras, Bcl2L11, CDKN1B and Zeb2.

**Conclusion:**

*In vivo* metastasis was confirmed in the modified Levrat’s model. Analysis of the primary tumor identified a distinctive miRNA signature for primary tumors that metastasized.

## Introduction

Surveillance Epidemiology and End Results Program (SEER) statistics show that approximately 34,000 people live with esophageal cancer in the United States[1–3]. The increased incidence and histologic change from squamous cell carcinoma to adenocarcinoma for esophageal cancer over the past four decades is one of the most dramatic changes observed in the history of human cancer[3]. Despite recent advances in multimodality therapy incorporating radiation, surgery, chemotherapy and newer biologic agents, the outcomes are still dismal (five-year survival of less than 20%)[4,5]. Therefore, there is a need to better understand the aspects of tumor biology that predict clinical behavior and identify novel molecular targets for therapy.

Previous studies have focused on identifying protein biomarkers of esophageal adenocarcinoma (EAC) to help predict tumor behavior and treatment response [6]. There has been an increased interest in non-coding RNAs (ncRNA) and microRNAs (miRNAs) and their potential use as indicators of cancer behavior. miRNA expression patterns have been identified for different tumor types [7] and are now known to play important roles in tumor development and associated pathways [8]. These expression patterns are thought to have potential roles as biomarkers, predictors of tumor response, and/or potential treatment targets [9–11]. However, most of the literature associated with esophageal cancer has been with respect to miRNA expression profiles of esophageal squamous cell carcinoma (ESCC) [7,12,13]. The predominant form of esophageal cancer in the United States and Europe is now adenocarcinoma [14].

The modified Levrat surgical model, which uses an end-to-side esophagojejunal anastomosis, has been used to study EAC. Previous studies have shown that the resultant gastroduodenojejunal reflux leads to a reliable progression from Barrett’s esophagus to esophageal adenocarcinoma on a histologic and molecular level [15]. The Levrat animal model is highly efficient for inducing tumorigenesis, with an observed 70% rate of adenocarcinoma development at 28 weeks after surgery. However, utilization of this model has been limited by the inability to demonstrate metastatic disease [16].

The objectives of the present study were to validate the Levrat model as an *in vivo* model of EAC metastasis and to identify a miRNA signature for EAC that is likely to metastasize using comparative miRNA analysis.

## Materials and Methods

### Ethics Statement

The Institutional Animal Care and Use Committee (IACUC) at University of Pittsburgh and the IACUC at Allegheny Health Network approved the respective study protocols, all animals used in this study were cared for, and all procedures were in compliance with the “Guide for the Care and Use of Laboratory Animals”. All animals were euthanized by carbon dioxide inhalation.

### Experimental Design

**Fig 1:**
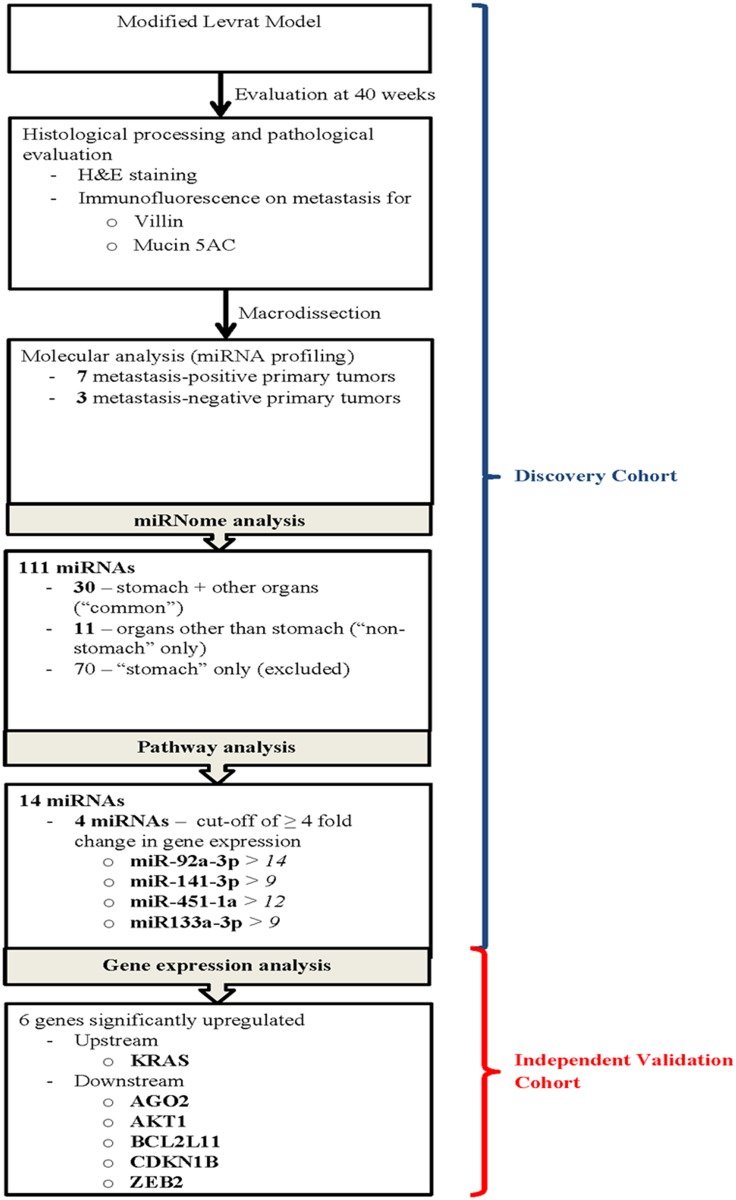
Study schema outlining the major steps in the experimental design and miRNA analysis.

Study schema outlining the major steps in the experimental design and miRNA analysis are represented in Fig. 1.

### Levrat Model

The Levrat model was used to create a surgical end-to-side esophagojejunal anastomosis in 6–8 week-old, 300 g male Sprague-Dawley rats (Harlan Laboratories, Indianapolis, IN) as previously described [17]. The animals were closely monitored post-operatively and weighed weekly. All animals were euthanized at 40 weeks post-operatively by carbon dioxide inhalation. If rats experienced a 25% or greater weight loss after immediate post-operative period or fit alternate euthanasia criteria, they were sacrificed prior to 40 weeks.

### Histological Processing and Pathological Evaluation

Upon necropsy, the entire esophagus and jejunum, to a length approximately 1 cm distal to anastomosis, was collected and opened longitudinally. The esophagus and jejunum were inspected visually. Lung, liver, stomach, and regional lymph nodes were collected from all animals at necropsy. Collected tissue specimens were snap frozen in Tissue-Tek O.C.T. compound (Sakura Finetek, Torrance, CA; #4583), sectioned into 5 μm slides, and stained with hematoxylin and eosin (H&E). The H&E stained slides were reviewed by two blinded pathologists. EAC was characterized by mucinous, dysplastic glandular cell growth with atypical nuclei and invasion through the basement membrane.

### Immunofluorescent Labeling (IF)

Immunofluorescent labeling using villin (VIL1) and mucin 5AC (MUC5AC) antibodies was performed on primary tumor from the esophagus to establish the presence of adenocarcinoma and on metastatic tissue to establish esophageal origin. Briefly, 5 μm frozen sections were fixed in 3.7% formaldehyde for 15 minutes followed by 3 washes of Tris-buffered saline (TBS). Non-specific antibody-epitope sites were blocked with 1% Bovine Serum Albumin (BSA)/5% goat serum in TBS for 1 hour. Incubation with the primary antibody or isotype control was performed for 1 hour followed by 3 TBS washes. MUC5AC (Pierce Biotechnology, Rockford, IL; #MA1-2907) was used at a 50x dilution, VIL1 (Thermo Fisher, Fremont, CA; #MS1499PO) at 2ug/ml. Secondary antibody incubation for 1 hour was performed using 2.5 ug/ml goat anti mouse Alexa 488 (Life Technologies, Carlsbad, CA; #A11017) and 5 ug/ml goat anti mouse Alexa 594 (Life Technologies, Carlsbad, CA; # A11020) for MUC5AC and VIL1, which was followed by 3 TBS washes. Slides were rinsed in water and allowed to dry. One drop of Prolong Gold with 4', 6-diamidino-2-phenylindole (DAPI) (Molecular Probes, Eugene, OR; #P36935) was added to each slide. Slides were cured overnight at room temperature in the dark and stored at 4°C.

Optimization and reproducibility of both stains were conducted before analysis of study samples. Intensity standardization of the immunofluorescent stains involved special attention to incubation times and co-interpretation of standard slides with positive, negative and isotype controls. Control tissues for VIL1 were kidney (positive control), normal esophagus (negative control) and mouse IgG on kidney (isotype control). Control tissues for MUC5AC were gastric epithelium (positive control), normal colon (negative control) and mouse IgG on gastric epithelium (isotype control). Positive villin staining was identified by sharp localized fluorescent signals in the brush border and cytoplasm of goblet and columnar cells. Mucin 5AC positive staining was identified by localized cytoplasmic fluorescent signal in mucus cells and goblet cells.

### Molecular Analyses

#### Macrodissection

Tumor tissue was macrodissected from primary esophageal specimens for molecular analyses. Fifteen 20 μm sections were cut in a cryostat. The tumor mass was dissected using a cold, RNase-free razor and placed in QIAzol buffer (Qiagen, Valencia, CA; #79306). The same number of sections were cut from normal esophageal specimens and placed directly into QIAzol buffer. RNA containing the miRNA population was isolated using the miRNeasy Kit (Qiagen, Valencia, CA; #217004). RNA concentration was spectrophotometrically assessed on the SpectraMax M2e plate reader (Molecular Devices, Sunnyvale, CA) and RNA quality was assessed by Bioanalyzer (Agilent, Santa Clara, CA) RIN (RNA Integrity Number).

#### miRNA Profiling

Three metastasis negative and 7 metastasis positive primary tumors were selected for miRNA profiling using 3216Z array (SA Bioscience, Frederick, MD). Briefly, 10 ng of total RNA containing miRNA was reverse transcribed in a total volume of 10ul at 37°C for 1 hour followed by inactivation of reverse transcriptase at 95°C for 5 minutes using the miScript II RT Kit (Qiagen, Valencia, CA; #218160) according to manufacturer recommendations. cDNA was diluted 5-fold in nuclease free water and 5 ul was preamplified using the miScript PreAmp Kit (Qiagen, Valencia, CA; #331451). Cycling parameters for preamplification were: 95°C for 15 minutes followed by 2 cycles of 94°C for 30 seconds, 55°C for 1 minute, 70°C for 1 minute followed by 10 cycles of 94°C for 30 seconds, 60°C for 3 minutes. Preamplified cDNA was diluted 20-fold in nuclease free water. PCR was performed on 100ul of preamplified cDNA using the miRNome miScript miRNA 3216Z PCR Array and the miScript SYBR Green PCR Kit (Qiagen, Valencia, CA; #218073). Cycling parameters for PCR were: 95°C for 15 minutes followed by 40 cycles of 94°C for 15 seconds, 55°C for 30 seconds, and 70°C for 30 seconds. Data was normalized to 5 endogenous control miRNAs: SNORD68, 72, 95, 96A and RNU6-2 and expression calculated by the delta-delta-Ct (2^-ΔCT^) method. The top differentially expressed miRNAs were identified using miScript miRNA PCR Array Data Analysis using a threshold of > 2 fold change and a p-value <0.05 (“focus miRNAs”) for significance. The results of the miRNA profiling were labeled by metastasis location: stomach or other organs. The filtering produced a list of 111 focus miRNAs: (1) 30 focus miRNAs were present in stomach and present in other organs (“common to all organs”); (2) 70 focus miRNAs were present in stomach only (“specific to stomach”); and (3) 11 focus miRNAs were present in distant sites except stomach (“specific to non-stomach organs”).

#### Pathway Analysis of miRNome

Two groups of differentially regulated focus miRNAs were analyzed by Ingenuity Pathway Analysis (IPA) to determine a miRNA signature for EAC metastasis: 1) 30 miRNAs common to all metastases and 2) 11 miRNAs specific to non-stomach organs. miRNAs that were specific to stomach metastases were not included in the analysis because of the possibility of “drop metastases” i.e., tumor cells that spread from the primary lesion site to the stomach via gastrointestinal fluid [18].

Focus miRNAs from the 2 groups were mapped to the IPA Knowledge Base to generate molecular networks displaying the interactions between miRNAs on a molecular level. IPA recognizes the focus miRNAs from the dataset and uses Qiagen’s Gene Ontology to assign a gene symbol name before running the gene expression analysis. The gene symbol name is determined by the miRNA’s canonical “seed sequence” and nucleotides 2–8 at the 5’ end of the miRNA, which is known to be important in mRNA target recognition across multiple organisms [19]. IPA ranks networks by interconnectivity and number of focus miRNAs, with the underlying assumption that more highly interacting miRNAs more likely represent significant biological function [20,21]. Networks are scored by the number of focus miRNAs[22]. For the group “common to all metastases,” the 2 top-scoring networks were “Cancer, Organismal Injury and Abnormalities, Reproductive System Disease” (IPA score 26, focus miRNAs 11) and “Cellular Growth, Proliferation, Cell Cycle, Developmental Disorder” (IPA score 20, focus miRNAs 9). For the group “specific to other organs,” the top-scoring network was “Cellular Development, Cellular Growth and Proliferation, Developmental Disorder” (IPA score 9, focus molecules 4). The 23 focus miRNAs from the top-scoring networks were selected for further analysis.

Canonical pathways for metastasis and upper gastrointestinal tract cancer were overlaid on the top-scoring networks to further identify the functional subset of 14 focus miRNAs over-represented in the neoplasia and metastasis literature. Finally, a cut-off of ≥ 4 fold change in gene expression was used to identify the final 4 focus miRNAs constituting the miRNA signature.

A functional analysis was performed on upstream signaling molecules and downstream targets of the 4 miRNAs to identify molecules in the top scoring networks related to neoplasia and metastasis. One upstream molecule (KRAS) and 6 downstream targets (AGO2, AKT1, BCL2, BCL2L11, CDKN1B, and ZEB2) were selected for further gene expression validation.

#### Gene Expression Validation

Gene expression analysis of mRNA upstream and downstream targets identified by the IPA analysis was performed on a secondary cohort of 5 normal esophagus control specimens, 5 metastasis positive, and 23 metastasis negative primary EAC samples. Total RNA was isolated as previously described from the macrodissected tumor. RNA for the control tissue was isolated from the entire section. Briefly, 500 ng and 125 ng of total RNA was reversed transcribed using the RT^2^ First Strand Kit (Qiagen, Valencia, CA #330401). For each sample, a no RT reaction (NRT) containing everything except the RT enzyme was performed. SYBRgreen PCR of 4.5 ng (1x input) and 1.125ng (4x input) cDNA as well as the NRT reaction and a no template control (NTC) was performed using the following RT^2^ Primer Assays: KRAS (Qiagen, Valencia, CA #PPR47860F), Ago2 (Qiagen, Valencia, CA #PPR48846A), Akt1 (Qiagen, Valencia, CA #PPR45425C), Bcl2 (Qiagen, Valencia, CA #PPR06577B), Bcl2L11 (Qiagen, Valencia, CA #PPR06472A), CDKN1B (Qiagen, Valencia, CA #PPR06391A), ZEB2 (Qiagen, Valencia, CA #PPR50195A), and control Rplp1 (Qiagen, Valencia, CA # PPR42363C). Cycling parameters were: 95°C for 10 minutes, 40 cycles of 95°C for 15 seconds, 60°C for 1 minute followed by dissociation curve analysis of 95°C for 15 seconds, 60°C for 1 minute, and 95°C for 15 seconds to show amplification of the specific amplicon. Ct values were generated and a 2 cycle difference between the 1x and 4x input was used to assess the quantitative nature of the RT reaction. Data was normalized to ribosomal protein, large, P1 (Rplp1) and relative expression values were calculated using the 2^-ΔCT^ method. The NRT reactions were used to show the absence of gDNA amplification and the NTC reactions to show absence of contamination.

### Statistical Analysis

Statistical analyses were performed using SPSS software (IBM, Armonk, NY, Version 20). A p-value < 0.05 was considered statistically significant. Independent two-tailed T-test were used to compare miRNA and gene expression profiles of metastasis positive primary tumors vs. metastasis negative primary tumors. For miRNA pathway analysis IPA software was used[23].

## Results

### Modified Levrat Rat Model

**Fig 2:**
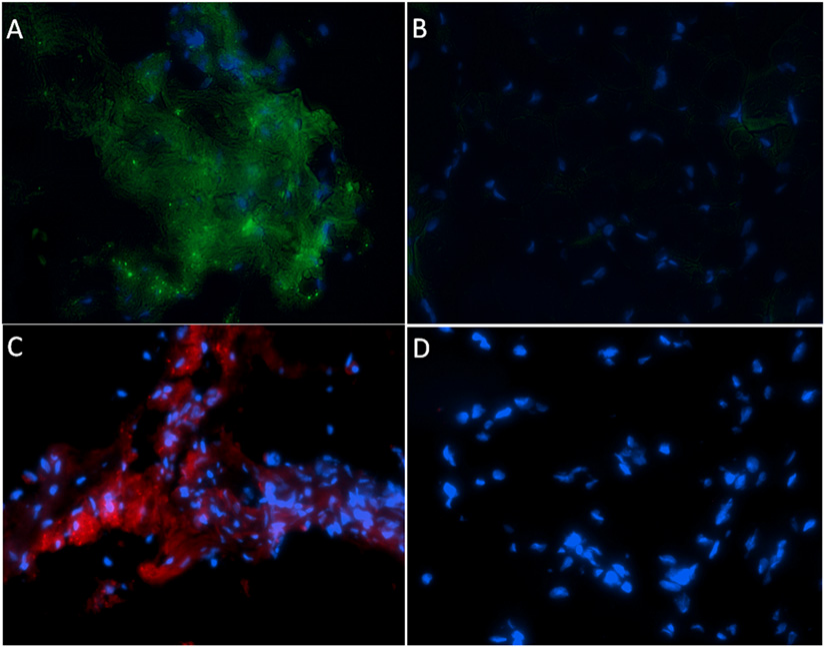
Immunofluorescence staining of rat tissue with MUC5AC and villin. Panel A and B show MUC5AC immunofluorescence staining for primary adenocarcinoma in Levrat esophagus and normal esophagus, respectively. Panel C and D show villin immunofluorescence staining in Levrat esophagus and normal esophagus, respectively. Positive MUC5AC and villin staining were detected in primary tumor with the Alexa Fluor 488 secondary antibody, conjugated to a green fluorophore and the Alexa 594 secondary antibody, conjugated to a red fluorophore, respectively. Normal esophagus shows the absence of MUC5AC and villin staining.

**Fig 3:**
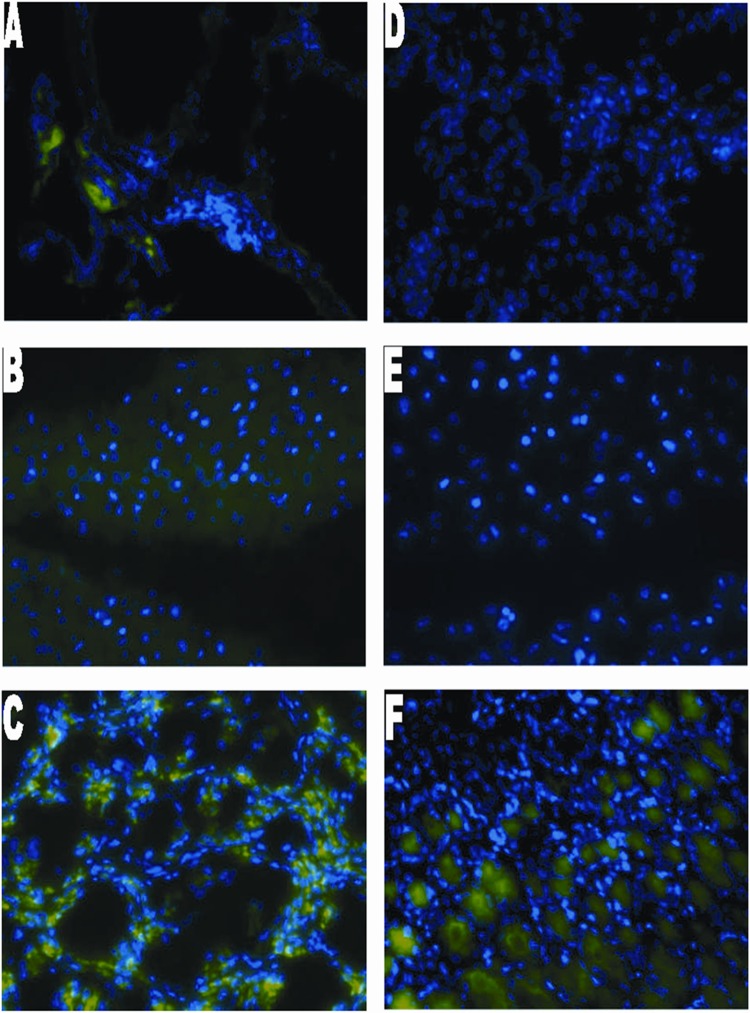
Immunofluorescence staining of rat tissue with MUC5AC. Panel A to F show immunofluorescence staining for metastatic lung, metastatic liver, metastatic stomach, non-metastatic lung, non-metastatic liver and non-metastatic stomach, respectively for representative cases. Positive MUC5AC cytoplasmic staining was detected in all metastatic samples with the Alexa Fluor 488 secondary antibody, conjugated to a green fluorophore. Metastasis negative liver and lung show the absence of MUC5AC staining. However, metastasis negative stomach stains positive for MUC5AC as gastric mucin M1 antigen is found in mucus cells of gastric epithelium.

**Fig 4:**
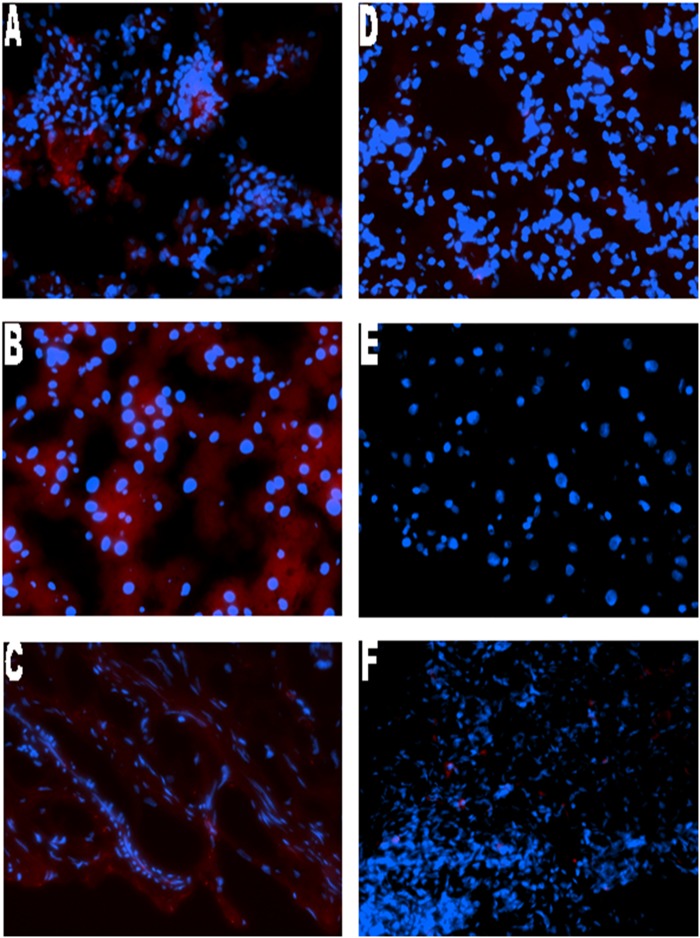
Immunofluorescence staining of rat tissue with villin. Representative case of (A)metastatic lung, (B) metastatic liver, (C) metastatic stomach, (D)non-metastatic lung, (E) non-metastatic liver and (F) non-metastatic stomach. Positive villin cytoplasmic staining was detected in all metastatic samples with the Alexa Fluor 594 secondary antibody, conjugated to a red fluorophore. Metastasis negative liver, lung and stomach show the absence of villin staining.

Forty-one total animals were included in the present study by undergoing esophagojejunostomy as described above. Twenty-nine rats survived to 40 weeks (29% mortality rate). Twelve rats died (2-died of unknown cause, 7-suffered post-operative anastomotic leak, 1-died of respiratory complications, and 2- sacrificed for persistent weight loss and found to have anastomotic stricture on necropsy). Of the 29 rats that survived to 40 weeks, 3 were tumor free, and 4 had a lesion with unclear pathology findings and were excluded. Twenty-two animals had adenocarcinoma confirmed on pathology of which 13 had metastatic disease. Epithelial origin of metastasis was validated by IF using villin and mucin 5AC (Figs. 2–4)

### miRNome Analysis

Four animals in the metastasis positive and 4 animals in the metastasis negative group had reverse-transcriptase inhibition during miRNome analysis and were discarded from the data set. Tumors from 7 of the metastasis positive animals and tumors from 3 of the metastasis negative animals were selected for miRNA profiling (S1 Fig.). The selected 7 metastasis positive animals had confirmed macro-metastases in the lung (1), liver/stomach (2), lymph node/stomach (1), and stomach (3), respectively.

Based on the comparative miRNome results, miScript miRNA PCR Array Data Analysis (Qiagen) identified differentially expressed miRNAs (“focus miRNAs”). 70 focus miRNAs were specific to stomach mets, 11 focus miRNAs were specific to other organ mets (lymph node, lung, liver), and 30 focus miRNAs were common to stomach mets and mets to other organs (Tables 1–2).

**Table 1:** Thirty differentially expressed miRNAs common to all organs (stomach and other distant sites).

Mature ID	Symbol	Fold Regulation	p value
**rno-miR-33-5p**	miR-33-5p (and other miRNAs w/seed UGCAUUG)	**-65.07**	**0.001**
**rno-miR-32-5p**	miR-92a-3p (and other miRNAs w/seed AUUGCAC)	**-29.05**	**0.001**
**rno-miR-141-3p**	miR-141-3p (and other miRNAs w/seed AACACUG)	**-18.01**	**0.006**
**rno-miR-29b-3p**	miR-29b-3p (and other miRNAs w/seed AGCACCA)	**-14.93**	**0.007**
**rno-miR-101a-3p**	miR-101-3p (and other miRNAs w/seed ACAGUAC)	**-13.55**	**0.026**
**rno-miR-326-3p**	miR-330-5p (and other miRNAs w/seed CUCUGGG)	**-11.88**	**0.001**
**rno-miR-96-5p**	miR-96-5p (and other miRNAs w/seed UUGGCAC)	**-11.77**	**0.017**
**rno-miR-24-2-5p**	miR-24-1-5p (and other miRNAs w/seed UGCCUAC)	**-10.22**	**0.038**
**rno-miR-1-5p**	miR-1-5p (miRNAs w/seed CACAUAC)	**-10.08**	**0.011**
**rno-miR-147**	miR-147 (and other miRNAs w/seed UGUGCGG)	**-7.43**	**0.003**
**rno-miR-19a-3p**	miR-19b-3p (and other miRNAs w/seed GUGCAAA)	**-7.3**	**0.004**
**rno-miR-345-5p**	miR-345-5p (miRNAs w/seed GCUGACC)	**-6.67**	**0.001**
**rno-miR-675-5p**	miR-675-5p (and other miRNAs w/seed GGUGCGG)	**-6.32**	**0.033**
**rno-miR-219a-5p**	miR-219a-5p (and other miRNAs w/seed GAUUGUC)	**-5.57**	**0.002**
**rno-miR-301b-3p**	miR-130a-3p (and other miRNAs w/seed AGUGCAA)	**-4.95**	**0.031**
**rno-miR-142-5p**	miR-142-5p (and other miRNAs w/seed AUAAAGU)	**-4.9**	**0.001**
**rno-miR-20b-5p**	miR-17-5p (and other miRNAs w/seed AAAGUGC)	**-4.86**	**0.003**
**rno-let-7i-3p**	let-7i-3p (miRNAs w/seed UGCGCAA)	**-4.74**	**0.032**
**rno-miR-193-3p**	miR-193a-3p (and other miRNAs w/seed ACUGGCC)	**-4.66**	**0.003**
**rno-miR-106b-5p**	miR-17-5p (and other miRNAs w/seed AAAGUGC)	**-4.5**	**0.011**
**rno-miR-200a-3p**	miR-141-3p (and other miRNAs w/seed AACACUG)	**-3.78**	**0.018**
**rno-miR-497-5p**	miR-16-5p (and other miRNAs w/seed AGCAGCA)	**-3.62**	**0.006**
**rno-miR-331-3p**	miR-331-3p (miRNAs w/seed CCCCUGG)	**-3.52**	**0.048**
**rno-miR-425-5p**	miR-425-5p (and other miRNAs w/seed AUGACAC)	**-3.32**	**0.017**
**rno-miR-130a-3p**	miR-130a-3p (and other miRNAs w/seed AGUGCAA)	**-3.15**	**0.002**
**rno-miR-30d-3p**	miR-30a-3p (and other miRNAs w/seed UUUCAGU)	**-2.93**	**0.003**
**rno-miR-17-5p**	miR-17-5p (and other miRNAs w/seed AAAGUGC)	**-2.73**	**0.025**
**rno-miR-20a-5p**	miR-17-5p (and other miRNAs w/seed AAAGUGC)	**-2.52**	**0.036**
**rno-miR-30b-5p**	miR-24-1-5p (and other miRNAs w/seed UGCCUAC)	**-2.05**	**0.022**
**rno-miR-340-3p**	miR-340-3p (and other miRNAs w/seed CCGUCUC)	**2.87**	**0.002**

A two-tailed, two-sample equal variance T-test was performed to obtain the p-values.

**Table 2:** Eleven differentially expressed miRNAs specific to distant organs excluding stomach.

Mature ID	Symbol	Fold Regulation	p value
**rno-miR-3561-5p**	miR-3561-5p (miRNAs w/seed CUGUGUC)	**-12.67**	**0.043**
**rno-miR-451-5p**	miR-451a (and other miRNAs w/seed AACCGUU)	**-12.58**	**0.018**
**rno-miR-133b-3p**	miR-133a-3p (and other miRNAs w/seed UUGGUCC)	**-9.33**	**0.03**
**rno-miR-133a-3p**	miR-133a-3p (and other miRNAs w/seed UUGGUCC)	**-7.89**	**0.041**
**rno-miR-540-5p**	miR-540-5p (miRNAs w/seed AAGGGUC)	**-6.28**	**0.001**
**rno-miR-296-5p**	miR-296-5p (miRNAs w/seed GGGCCCC)	**-6.25**	**0.037**
**rno-miR-874-5p**	miR-874-5p (miRNAs w/seed GGCCCCA)	**-5.25**	**0.03**
**rno-miR-376a-3p**	miR-376a-3p (miRNAs w/seed UCGUAGA)	**-3.23**	**0.011**
**rno-miR-429**	miR-200b-3p (and other miRNAs w/seed AAUACUG)	**-2.13**	**0.018**
**rno-miR-24-1-5p**	miR-30c-5p (and other miRNAs w/seed GUAAACA)	**-2.05**	**0.033**
**rno-miR-489-5p**	miR-489-5p (miRNAs w/seed GUCGUAU)	**-2.00**	**0.003**

A two-tailed, two-sample equal variance T-test was performed to obtain the p-values.

### Pathway Analysis of miRNome

IPA identified 4 focus miRNAs related to metastasis and upper gastrointestinal tract cancer (≥4 fold change) that were downregulated in metastasis positive samples versus metastasis negative samples: 1) miR-92a-3p (p = 0.0001, fold change >14), miR-141-3p (p = 0.0022, fold change >9), miR-451-1a (p = 0.0181, fold change >12) and miR133a-3p (p = 0.0304, fold change >9) (S2 Fig.). The 4 focus miRNAs symbols miR-92a-3p, miR-141-3p, miR-451-1a, and miR133a-3p were mapped from the dataset ID rno-miR-32-5p, rno-miR-141-3p, rno-miR-451-5p, and rno-miR-133b-3p respectively.

**Fig 5:**
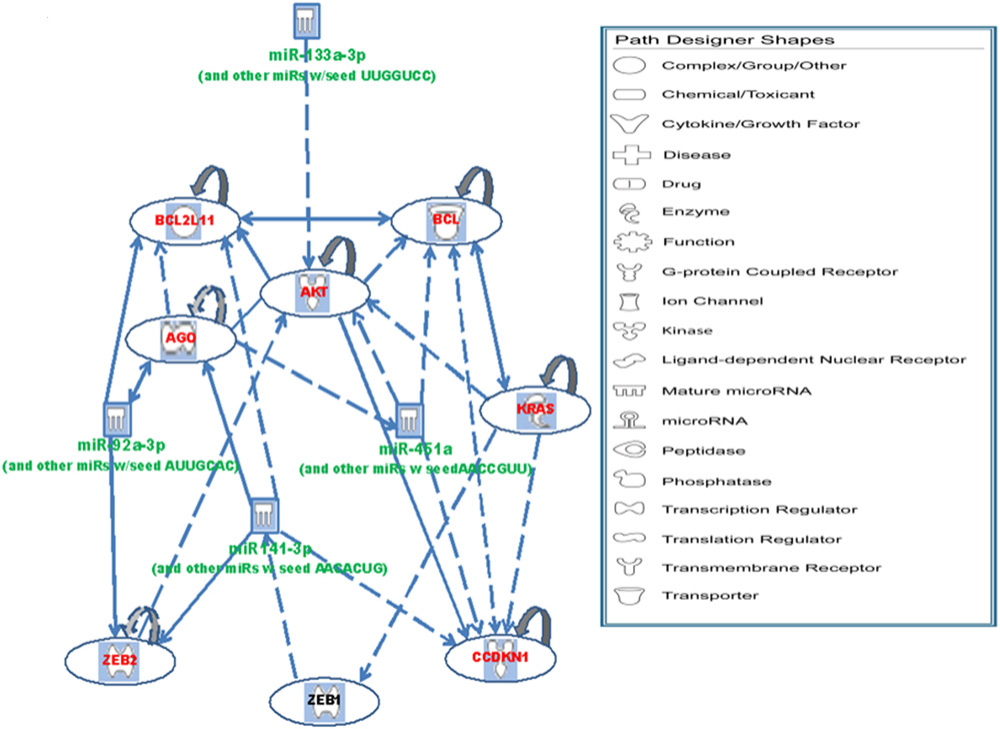
Cellular interactions between miRNAs and target genes. Green represents downregulation and red represents upregulation of gene expression. A two-tailed, two-sample equal variance T-test was performed to obtain the p-values.

To determine if the selected miRNA signature would predict significant changes in expression of genes within the canonical neoplastic and metastasis pathways, IPA was used to identify 1 upstream negative regulator (KRAS) of miR-141-3p and 6 downstream, positively regulated targets (AGO2, BCL2L11, AKT1, ZEB2, CDKN1B, BCL2) of the 4 miRNAs. The interactions between the miRNAs and upstream regulator/downstream targets are summarized in Fig. 5.

IPA identified the top 5 canonical pathways associated with the miRNA signature and downstream molecules as PTEN signaling (p-value 3.64E-09), prostate cancer signaling (p-value 1E-07), pancreatic adenocarcinoma signaling (p-value 2.83E-07), PI3K/AKT signaling (p-value 5.15E-07), and molecular mechanisms of cancer (p-value 1.02E-6) (S3 Fig., S1 Table). The results of the IPA analysis selection for the miRNA signature, upstream/downstream targets, and associated biological function are summarized in Table 3 (S2 Table).

**Table 3:** Target genes of the miRNA signature and associated cell function/disease processes.

	miRNA regulation—Downstream Targets					
**Positively Regulated Downstream Targets**	miR-92a-3p	miR-141-3p	miR-451-a	miR-133a-3p	**Function in cell**	**Mediated disease processes**
*AGO2*	✔	✔	✔		RNA interference, gene silencing	Epithelial neoplasia [24], `esophageal cancer [24]
*AKT1*			✔	✔	Cell growth, survival	Epithelial neoplasia[25], metastasis[26]
*BCL2*			✔		Suppressor of apoptosis	Epithelial neoplasia [27], tumorigenesis [28,29]
*BCL2L11*	✔	✔			Mediator of Apoptosis	Hematological neoplasia [30], hyperplasia [31]
*CDKN1B (p27)*			✔	✔	Cell cycle control	Epithelial neoplasia [29], tumorigenesis [32]
*Zeb2*	✔	✔			Regulator of growth and development	Epithelial neoplasia [33], tumorigenesis [34]
**Upstream Negative Regulators**	**miRNA regulation—Upstream Regulator**					
*KRAS*		✔			Regulates cell growth and survival	Tumorigenesis, metastasis[35]

### Gene Expression Analysis

**Fig 6:**
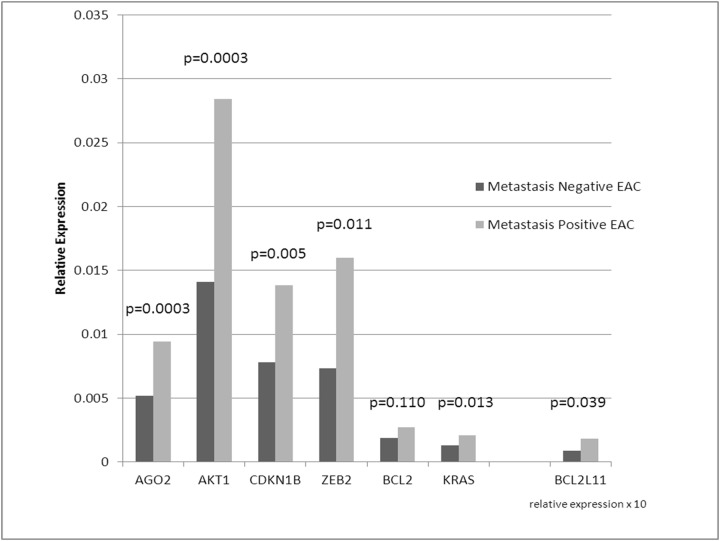
Relative gene expression levels in a secondary cohort of metastasis negative and metastasis positive primary EAC in the Levrat Model. Quantitative reverse transcription-PCR (QRT-PCR) was performed on seven gene targets identified by the metastatic miRNA signature in IPA. Six of seven target genes were significantly upregulated in primary tumors with metastasis.

To further confirm findings, relative gene expression analysis of 1 upstream and 6 downstream genes targeted by the miRNA signature was performed. Six of seven genes showed significant differential expression in primary EAC samples with metastases compared to without metastases (Fig. 6).

## Discussion

Results of the present study show for the first time metastatic disease in the Levrat model, and identify a miRNA profile associated with the presence of metastasis. Esophageal origin of metastases was confirmed by the presence of VIL1+/MUC5AC+ cells. As such, results of this study confirm the value of the Levrat model for the study of not only EAC, but also metastatic EAC.

Metastases formed spontaneously from the primary tumor, without the use of an external carcinogen or injection of a metastatic esophageal cancer cell line. Perhaps mimicking a clinical scenario of metastases, cancer cells from the primary rodent tumor were induced to migrate to distal sites with longer survival times and sustained exposure to the reflux. Other animal models of EAC have relied upon a non-physiological carcinogen such as 2, 6,-dimethylnitrosomorphine (2, 6-DMNM) to induce EAC tumorigenesis [36]. Models of metastasis involving injection of metastatic cancer cells intracardially or intravenously risk obscuring translatable insights into the biology of metastasis by introducing genetic variation during cell culture [37].

The Levrat surgical model has been previously reported by other groups to present reliable progression of Barrett’s Esophagus and EAC by 28 weeks [17], 36 weeks [38], and 40 weeks [39]. However, metastasis to distant organs has not been observed. According to the traditional model of metastasis, the invasion-metastasis cascade is a late-acquired event in tumorigenesis [37]. Later time points, such as the 40 week time point used in the present study, may be required for the micro- and macro-metastases to be detected in the Levrat model.

One example of the Levrat animal model of metastasis for clinical use, as shown in the present study, is the identification of primary tumor biomarkers that predict the likelihood of metastatic disease. Emerging classes of tumor biomarkers are the miRNAs—small, non-coding RNAs that regulate gene expression via degradation or translational inhibition of target mRNAs. miRNAs make attractive cancer biomarkers because their expression is known to be differentially regulated in normal tissue versus neoplastic tissue and may be globally profiled using high-throughput microarrays [40,41]. Recently, miRNAs have been found to have a direct, non-coding role in tumor suppression by targeting several oncogenes involved in specific cancer-related pathways [42].

There is a profound need for an animal model of EAC metastasis. Individuals with EAC face grim prospects: 50% of patients will have metastatic disease before the time of diagnosis. Therefore, a representative preclinical model is vital for progress to be made in EAC research. Despite screening procedures and novel approaches for the treatment of high-grade dysplasia and EAC such as radiofrequency ablation [43] and stepwise radical endoscopic resection [44], mortality rates remain high secondary to poor risk stratification leading to late discovery. There are substantial limitations to current treatments including the need for recurrent interventions, risk of stricture formation, and the development of metachronous lesions and/or metastatic disease. EAC still poses a number of challenges secondary to its natural history despite improvements in minimally invasive surgical techniques, which successfully decrease hospital stays and perioperative morbidity,

The most common sites of EAC metastases observed clinically include liver, lung, and lymph nodes [45,46]. Macro-metastases in the present study occurred in the lung (1), liver/stomach (2), lymph node/stomach (1), and stomach (3). Stomach metastases are not a reported site of EAC metastasis and for this reason were excluded as a group from the gene pathway analysis. It is plausible that the stomach metastases found in this study were “drop metastases” or cancer cells that had been shed from the primary tumor and spread to the stomach via the peritoneal cavity or esophageal lumen. Only metastases established through clinically relevant processes via the blood and lymphatic systems were studied [47].

In the present study, the miRNome analyses were conducted using 7 metastatic (experimental group) and 3 non-metastatic (control) animals with primary EAC. The large miRNA datasets were filtered using IPA analysis to identify a smaller subset of miRNAs in pathways common to the metastatic sites, which in turn yielded the 4 miRNA signature of metastatic EAC.

This signature could be used to further characterize the molecular and genetic changes associated with metastatic neoplasia. The 4 miRNAs (miR-92a-3p, miR-451a, miR-141-3p, and miR-133-3p) were significantly down regulated in metastasis positive tumors. Five downstream (AGO2, BCL2L11, AKT1, ZEB2, CDKN1B) and 1 upstream targets of the miRNAs (KRAS) in the signature were significantly overexpressed in metastasis positive tumors. The downstream targets are associated with epithelial neoplasia and metastasis. The upstream target KRAS has been shown to activate Zeb1, a repressor of miR-141-3p[35]. Zeb1 and Zeb2 (shown to be overexpressed in the present study) are important transcription factors in the epithelial-mesenchymal transition, a process known to drive tumorigenesis and metastasis[48].

Recent studies have shown that the miRNA expression changes in human esophageal adenocarcinoma[49,50]. Feber et.al, showed changes in miR-100, 199a-3p, 199a-5p, miR-143 and miR-145. Although the miRNA signature is different from the miRNA signature shown in our study, the targeted genes overlap by TargetScan analysis. miR-100 targets AGO2, miR-199a-3p and miR-145 target ZEB2, miR-143 targets BCL2, and miR-199a-5p targets CDKN1B, which are 4 of 7 genes targeted by the rat miRNA signature in the present study. In a more recent paper by Chen et.al, 3 miRNAs were differentially expressed in human esophageal adenocarcinoma: miR-200a, miR-21, and miR-133a. MiR-200a targets ZEB2, and miR-21 targets BCL2. Whereas, miR-133b shares sequence homology and gene targets with miR-133a, which was identified as a part of the rat miRNA signature in the present study. Therefore, the rat miRNA profile described in the present study corroborates these results, and predicts similar gene targets, as described previously[49,50].

The top 5 canonical pathways associated with the miRNA signature and downstream/upstream molecules were PTEN signaling, prostate cancer signaling, pancreatic adenocarcinoma signaling, PI3K/AKT signaling, and molecular mechanisms of cancer. The PI3K/AKT and PTEN pathways play a central role in regulating cell growth and survival [51]. It is known that when cell proliferation increases, the chance of an oncogenic mutation increases, which may drive the multi-step process from neoplasia to metastasis [47]. Many of the key molecules of the PTEN and P13K/AKT pathways overlap with the molecular mechanisms of the cancer pathway. The identification of the prostate cancer signaling pathway and the pancreatic adenocarcinoma signaling pathway suggest the interactions between the miRNAs and downstream molecules may be similar to the progression of cancer in other organs.

Taken together, the down-regulation of 4 miRNAs affecting multiple molecules of the PI3K/AKT, PTEN, and the molecular mechanisms of cancer pathways suggest multiple neoplastic drivers contribute to cell invasiveness—the most aggressive, end-product of tumor formation.

Further study into the pathways noted may help identify novel therapeutic targets specific to EAC [52]. More importantly, from a clinical standpoint, a unique aspect of the present study is the fact that the miRNome analysis was conducted on the primary tumor itself which plausibly characterized an expression profile unique to primary tumors that had the propensity to metastasize. If metastatic potential is identified early at the time of diagnosis, it may potentially help modulate treatment in several ways. First, it may spare metastasis-unlikely patients from the cytotoxic effects of unnecessary chemotherapy/radiation when resection alone could be effective. Second, a predictive miRNA signature on the primary tumor could dictate an aggressive treatment regimen for metastasis- likely patients. The latter is the group of patients suited more for clinical trials with novel agents that may potentially modulate some of the pathways outlined.

Similar miRNA signatures to those identified in the present study have been shown to predict survival, as well as to stratify the relative risk of patients in esophageal squamous cell carcinoma and other types of neoplasia [12,53]. More studies on human tissues of the signature derived in the present study could reveal similar correlations.

The limitations of the present study include the sole use of an animal model and the small sample size. Further studies with a larger sample size are needed to validate the predictive nature of the reported miRNA signature. However, since the model is genetically conserved and we did validate the numerous linked genes, studies on human tissue would be a logical next step to corroborate findings of the present study. Such studies with human tissue would establish direct clinical relevance and would be important in validating therapeutic targets in the canonical pathways of tumorigenesis and metastasis.

In summary, the present study validated the Levrat model as an *in vivo* model of metastatic EAC and identified a unique miRNA signature for EAC metastasis within the primary tumor using comparative miRNA analysis. It is important to note that both the miRNA signature and the analysis were conducted on the primary tumors and not the metastatic sites. This has important implications in identifying and predicting primary tumors that have the potential and propensity to metastasize.

## Supporting Information

S1 Fig Selection of samples for miRNome analysis. (TIFF)

S2 Fig Screenshots of IPA analysis. (TIFF)

S3 Fig Top canonical pathways associated with the miRNA signature and profiled mRNAs. (TIFF)

S1 Table Top 5 canonical pathways for the 4 miRNA signature and associated downstream/upstream molecules. (PDF)

S2 Table Fold change in gene expression and p-value of the 4 miRNA signature and downstream/upstream targets. (PDF)

## References

[1] Bollschweiler E, Wolfgarten E, Gutschow C, Holscher AH (2001) Demographic variations in the rising incidence of esophageal adenocarcinoma in white males. Cancer 92: 549–555. 11505399 10.1002/1097-0142(20010801)92:3<549::aid-cncr1354>3.0.co;2-l

[2] Edgren G, Adami HO, Weiderpass E, Nyren O (2013) A global assessment of the oesophageal adenocarcinoma epidemic. Gut 62: 1406–1414. 10.1136/gutjnl-2012-302412 22917659

[3] Dubecz A, Solymosi N, Stadlhuber RJ, Schweigert M, Stein HJ, et al (2013) Does the Incidence of Adenocarcinoma of the Esophagus and Gastric Cardia Continue to Rise in the Twenty-First Century?-a SEER Database Analysis. J Gastrointest Surg. 10.1007/s11605-013-2345-8 24234242

[4] Rubenstein JH, Chen JW (2014) Epidemiology of gastroesophageal reflux disease. Gastroenterology clinics of North America 43: 1–14. 10.1016/j.gtc.2013.11.006 24503355

[5] Mariette C, Piessen G, Briez N, Gronnier C, Triboulet JP (2011) Oesophagogastric junction adenocarcinoma: which therapeutic approach? The lancet oncology 12: 296–305. 10.1016/S1470-2045(10)70125-X 21109491

[6] Hong L, Han Y, Zhang H, Zhao Q, Wu K, et al (2014) Prognosis-related microRNAs in esophageal cancer. Expert opinion on biological therapy 14: 483–489. 10.1517/14712598.2014.882896 24506707

[7] Li SQ, Li F, Xiao Y, Wang CM, Tuo L, et al (2014) Comparison of long noncoding RNAs, microRNAs and messenger RNAs involved in initiation and progression of esophageal squamous cell carcinoma. Molecular medicine reports. 10.3892/mmr.2014.2287 PMC4094766 24888564

[8] Croce CM (2009) Causes and consequences of microRNA dysregulation in cancer. Nat Rev Genet 10: 704–714. 10.1038/nrg2634 19763153 PMC3467096

[9] Jones KB, Salah Z, Del Mare S, Galasso M, Gaudio E, et al (2012) miRNA signatures associate with pathogenesis and progression of osteosarcoma. Cancer Res 72: 1865–1877. 10.1158/0008-5472.CAN-11-2663 22350417 PMC3328547

[10] Huang WC, Chan SH, Jang TH, Chang JW, Ko YC, et al (2014) miRNA-491-5p and GIT1 serve as modulators and biomarkers for oral squamous cell carcinoma invasion and metastasis. Cancer Res 74: 751–764. 10.1158/0008-5472.CAN-13-1297 24335959

[11] Yu S, Lu Z, Liu C, Meng Y, Ma Y, et al (2010) miRNA-96 suppresses KRAS and functions as a tumor suppressor gene in pancreatic cancer. Cancer Res 70: 6015–6025. 10.1158/0008-5472.CAN-09-4531 20610624

[12] Chen Z, Li J, Tian L, Zhou C, Gao Y, et al (2014) MiRNA expression profile reveals a prognostic signature for esophageal squamous cell carcinoma. Cancer letters 350: 34–42. 10.1016/j.canlet.2014.04.013 24769072

[13] Fu C, Dong W, Wang Z, Li H, Qin Q, et al (2014) The expression of miR-21 and miR-375 predict prognosis of esophageal cancer. Biochemical and biophysical research communications 446: 1197–1203. 10.1016/j.bbrc.2014.03.087 24680681

[14] Shaheen NJ (2005) Advances in Barrett's esophagus and esophageal adenocarcinoma. Gastroenterology 128: 1554–1566. 15887151 10.1053/j.gastro.2005.03.032

[15] Miyashita T, Shah FA, Miwa K, Sasaki S, Nishijima K, et al (2013) Impact of inflammation-metaplasia-adenocarcinoma sequence and prevention in surgical rat models. Digestion 87: 6–11. 10.1159/000343896 23343962

[16] Raggi M, Langer R, Feith M, Friess H, Schauer M, et al (2010) Successful evaluation of a new animal model using mice for esophageal adenocarcinoma. Langenbecks Arch Surg 395: 347–350. 10.1007/s00423-010-0607-4 20300770

[17] Gibson MK, Zaidi AH, Davison JM, Sanz AF, Hough B, et al (2013) Prevention of Barrett esophagus and esophageal adenocarcinoma by smoothened inhibitor in a rat model of gastroesophageal reflux disease. Ann Surg 258: 82–88. 10.1097/SLA.0b013e318270500d 23108119

[18] Blecker D, Abraham S, Furth EE, Kochman ML (1999) Melanoma in the gastrointestinal tract. Am J Gastroenterol 94: 3427–3433. 10606298 10.1111/j.1572-0241.1999.01604.x

[19] Lewis BP, Shih IH, Jones-Rhoades MW, Bartel DP, Burge CB (2003) Prediction of mammalian microRNA targets. Cell 115: 787–798. 14697198 10.1016/s0092-8674(03)01018-3

[20] Ravasz E, Somera AL, Mongru DA, Oltvai ZN, Barabasi AL (2002) Hierarchical organization of modularity in metabolic networks. Science 297: 1551–1555. 12202830 10.1126/science.1073374

[21] Spirin V, Mirny LA (2003) Protein complexes and functional modules in molecular networks. Proc Natl Acad Sci U S A 100: 12123–12128. 14517352 10.1073/pnas.2032324100 PMC218723

[22] Calvano SE, Xiao W, Richards DR, Felciano RM, Baker HV, et al (2005) A network-based analysis of systemic inflammation in humans. Nature 437: 1032–1037. 16136080 10.1038/nature03985

[23] Kramer A, Green J, Pollard J Jr, Tugendreich S (2014) Causal analysis approaches in Ingenuity Pathway Analysis. Bioinformatics 30: 523–530. 10.1093/bioinformatics/btt703 24336805 PMC3928520

[24] Yoo NJ, Hur SY, Kim MS, Lee JY, Lee SH (2010) Immunohistochemical analysis of RNA-induced silencing complex-related proteins AGO2 and TNRC6A in prostate and esophageal cancers. APMIS 118: 271–276. 10.1111/j.1600-0463.2010.02588.x 20402672

[25] Stemke-Hale K, Gonzalez-Angulo AM, Lluch A, Neve RM, Kuo WL, et al (2008) An integrative genomic and proteomic analysis of PIK3CA, PTEN, and AKT mutations in breast cancer. Cancer Res 68: 6084–6091. 10.1158/0008-5472.CAN-07-6854 18676830 PMC2680495

[26] Kim EK, Yun SJ, Ha JM, Kim YW, Jin IH, et al (2011) Selective activation of Akt1 by mammalian target of rapamycin complex 2 regulates cancer cell migration, invasion, and metastasis. Oncogene 30: 2954–2963. 10.1038/onc.2011.22 21339740

[27] Puhr M, Santer FR, Neuwirt H, Susani M, Nemeth JA, et al (2009) Down-regulation of suppressor of cytokine signaling-3 causes prostate cancer cell death through activation of the extrinsic and intrinsic apoptosis pathways. Cancer Res 69: 7375–7384. 10.1158/0008-5472.CAN-09-0806 19738059

[28] Pierce RH, Vail ME, Ralph L, Campbell JS, Fausto N (2002) Bcl-2 expression inhibits liver carcinogenesis and delays the development of proliferating foci. Am J Pathol 160: 1555–1560. 12000706 10.1016/S0002-9440(10)61101-7 PMC1850870

[29] Matsuda Y, Ichida T (2006) p16 and p27 are functionally correlated during the progress of hepatocarcinogenesis. Med Mol Morphol 39: 169–175. 17187177 10.1007/s00795-006-0339-2

[30] Shang Q, Zhang D, Guo C, Lin Q, Guo Z, et al (2012) Potential synergism of Bim with p53 in mice with Mycinduced lymphoma in a mouse lymphoma model. Mol Med Rep 5: 1401–1408. 10.3892/mmr.2012.844 22446994

[31] Bouillet P, Metcalf D, Huang DC, Tarlinton DM, Kay TW, et al (1999) Proapoptotic Bcl-2 relative Bim required for certain apoptotic responses, leukocyte homeostasis, and to preclude autoimmunity. Science 286: 1735–1738. 10576740 10.1126/science.286.5445.1735

[32] King TJ, Gurley KE, Prunty J, Shin JL, Kemp CJ, et al (2005) Deficiency in the gap junction protein connexin32 alters p27Kip1 tumor suppression and MAPK activation in a tissue-specific manner. Oncogene 24: 1718–1726. 15608667 10.1038/sj.onc.1208355

[33] Redova M, Svoboda M, Slaby O (2011) MicroRNAs and their target gene networks in renal cell carcinoma. Biochemical and Biophysical Research Communications 405: 153–156. 10.1016/j.bbrc.2011.01.019 21232526

[34] Karreth FA, Tay Y, Pema D, Ala U, Tan SM, et al (2011) In Vivo Identification of Tumor-Suppressive PTEN ceRNAs in an Oncogenic BRAF-Induced Mouse Model of Melanoma (vol 147, pg 382, 2011). Cell 147: 948–948. 10.1016/j.cell.2011.09.032 PMC3236086 22000016

[35] Liu Y, Sanchez-Tillo E, Lu X, Huang L, Clem B, et al (2014) The ZEB1 transcription factor acts in a negative feedback loop with miR200 downstream of Ras and Rb1 to regulate Bmi1 expression. J Biol Chem 289: 4116–4125. 10.1074/jbc.M113.533505 24371144 PMC3924277

[36] Attwood SE, Smyrk TC, DeMeester TR, Mirvish SS, Stein HJ, et al (1992) Duodenoesophageal reflux and the development of esophageal adenocarcinoma in rats. Surgery 111: 503–510. 1598670

[37] Weigelt B, Peterse JL, van 't Veer LJ (2005) Breast cancer metastasis: markers and models. Nat Rev Cancer 5: 591–602. 16056258 10.1038/nrc1670

[38] Sui G, Bonde P, Dhara S, Broor A, Wang J, et al (2006) Epidermal growth factor receptor and hedgehog signaling pathways are active in esophageal cancer cells from rat reflux model. J Surg Res 134: 1–9. 16488438 10.1016/j.jss.2005.12.029

[39] Pera M, Brito MJ, Poulsom R, Riera E, Grande L, et al (2000) Duodenal-content reflux esophagitis induces the development of glandular metaplasia and adenosquamous carcinoma in rats. Carcinogenesis 21: 1587–1591. 10910963

[40] Pacurari M, Addison JB, Bondalapati N, Wan YW, Luo D, et al (2013) The microRNA-200 family targets multiple non-small cell lung cancer prognostic markers in H1299 cells and BEAS-2B cells. Int J Oncol 43: 548–560. 10.3892/ijo.2013.1963 23708087 PMC3775564

[41] Jeffrey SS (2008) Cancer biomarker profiling with microRNAs. Nat Biotechnol 26: 400–401. 10.1038/nbt0408-400 18392022

[42] Ozen M, Creighton CJ, Ozdemir M, Ittmann M (2008) Widespread deregulation of microRNA expression in human prostate cancer. Oncogene 27: 1788–1793. 17891175 10.1038/sj.onc.1210809

[43] Keeley SB, Pennathur A, Gooding W, Landreneau RJ, Christie NA, et al (2007) Photodynamic therapy with curative intent for Barrett's esophagus with high grade dysplasia and superficial esophageal cancer. Ann Surg Oncol 14: 2406–2410. 17534685 10.1245/s10434-007-9392-x

[44] Sharma VK, Wang KK, Overholt BF, Lightdale CJ, Fennerty MB, et al (2007) Balloon-based, circumferential, endoscopic radiofrequency ablation of Barrett's esophagus: 1-year follow-up of 100 patients. Gastrointest Endosc 65: 185–195. 17258973 10.1016/j.gie.2006.09.033

[45] Hess KR, Varadhachary GR, Taylor SH, Wei W, Raber MN, et al (2006) Metastatic patterns in adenocarcinoma. Cancer 106: 1624–1633. 16518827 10.1002/cncr.21778

[46] Luketich JD, Friedman DM, Weigel TL, Meehan MA, Keenan RJ, et al (1999) Evaluation of distant metastases in esophageal cancer: 100 consecutive positron emission tomography scans. Ann Thorac Surg 68: 1133–1136; discussion 1136–1137. 10543468 10.1016/s0003-4975(99)00974-1

[47] Weinberg RA (2014) The Biology of Cancer New York, NY: Garland Science.

[48] Park SM, Gaur AB, Lengyel E, Peter ME (2008) The miR-200 family determines the epithelial phenotype of cancer cells by targeting the E-cadherin repressors ZEB1 and ZEB2. Genes Dev 22: 894–907. 10.1101/gad.1640608 18381893 PMC2279201

[49] Feber A, Xi L, Luketich JD, Pennathur A, Landreneau RJ, et al (2008) MicroRNA expression profiles of esophageal cancer. J Thorac Cardiovasc Surg 135: 255–260; discussion 260. 10.1016/j.jtcvs.2007.08.055 18242245 PMC2265073

[50] Chen Z, Saad R, Jia P, Peng D, Zhu S, et al (2013) Gastric adenocarcinoma has a unique microRNA signature not present in esophageal adenocarcinoma. Cancer 119: 1985–1993. 10.1002/cncr.28002 23456798 PMC3731210

[51] Porta C, Paglino C, Mosca A (2014) Targeting PI3K/Akt/mTOR Signaling in Cancer. Front Oncol 4: 64 10.3389/fonc.2014.00064 24782981 PMC3995050

[52] Lin ML, Lu YC, Chen HY, Lee CC, Chung JG, et al (2014) Suppressing the formation of lipid raft-associated Rac1/PI3K/Akt signaling complexes by curcumin inhibits SDF-1alpha-induced invasion of human esophageal carcinoma cells. Mol Carcinog 53: 360–379. 10.1002/mc.21984 23192861

[53] Blandino G, Fazi F, Donzelli S, Kedmi M, Sas-Chen A, et al (2014) Tumor suppressor microRNAs: A novel non-coding alliance against cancer. FEBS letters. 10.1016/j.febslet.2014.03.033 24681102

