# Supplementary figures and images for: MicroRNA Signature Characterizes Primary Tumors That Metastasize in an Esophageal Adenocarcinoma Rat Model
Source: PLoS One. 2015 Mar 31;10(3):e0122375. doi: 10.1371/journal.pone.0122375 (PMC4380408; doi:10.1371/journal.pone.0122375)

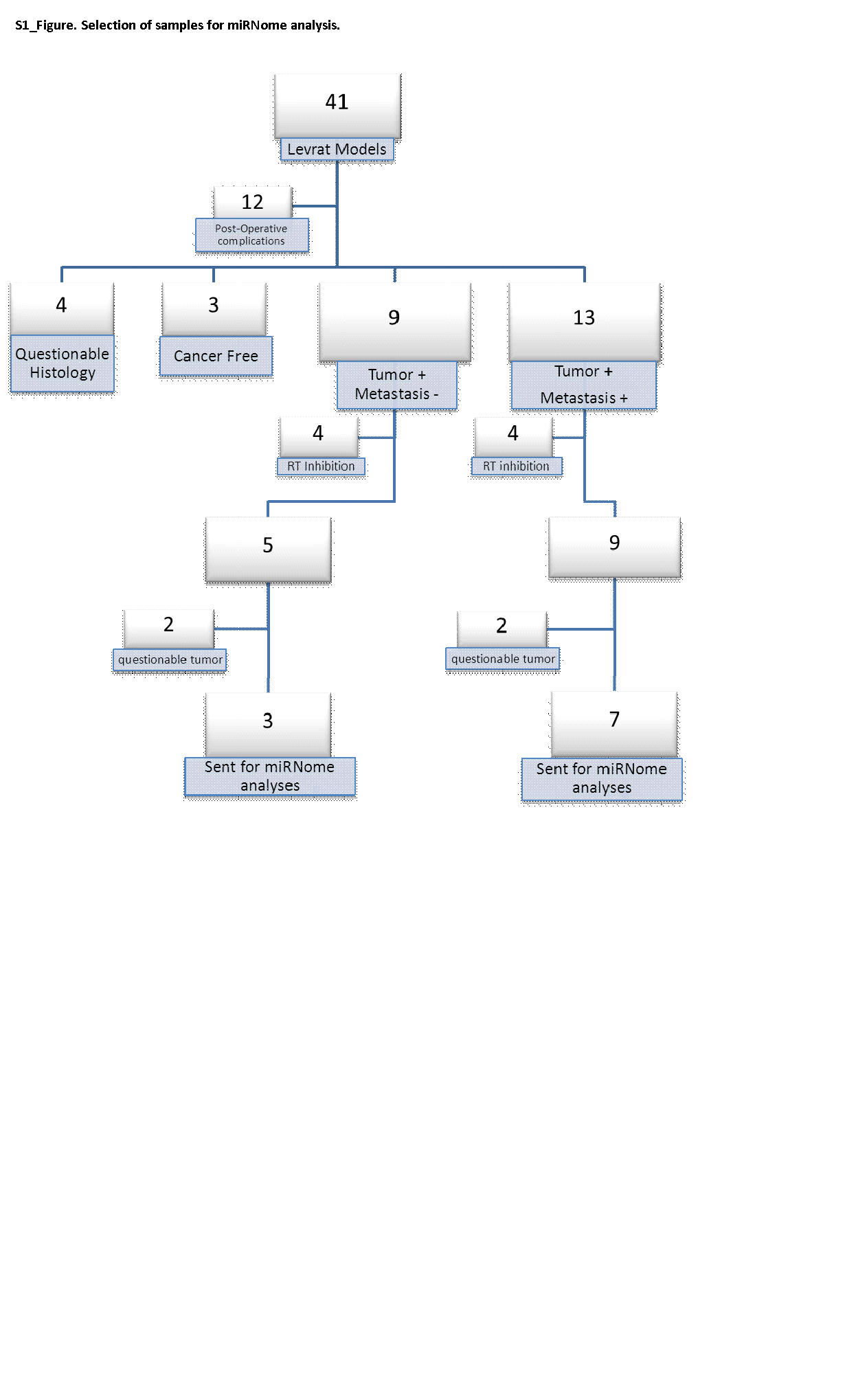

Supplement: S1 Fig — (TIFF) [file pone.0122375.s001.tiff]

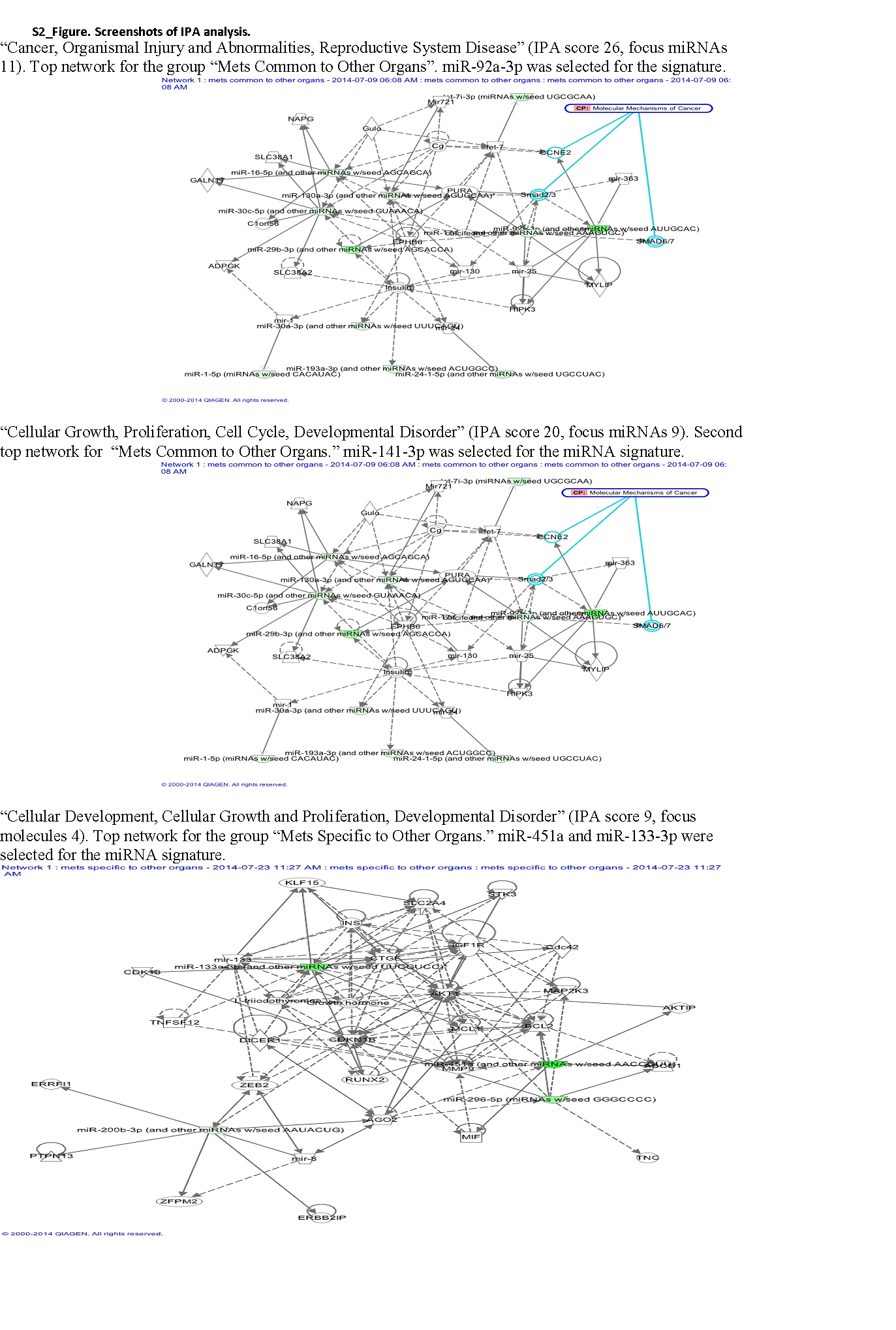

Supplement: S2 Fig — (TIFF) [file pone.0122375.s002.tiff]

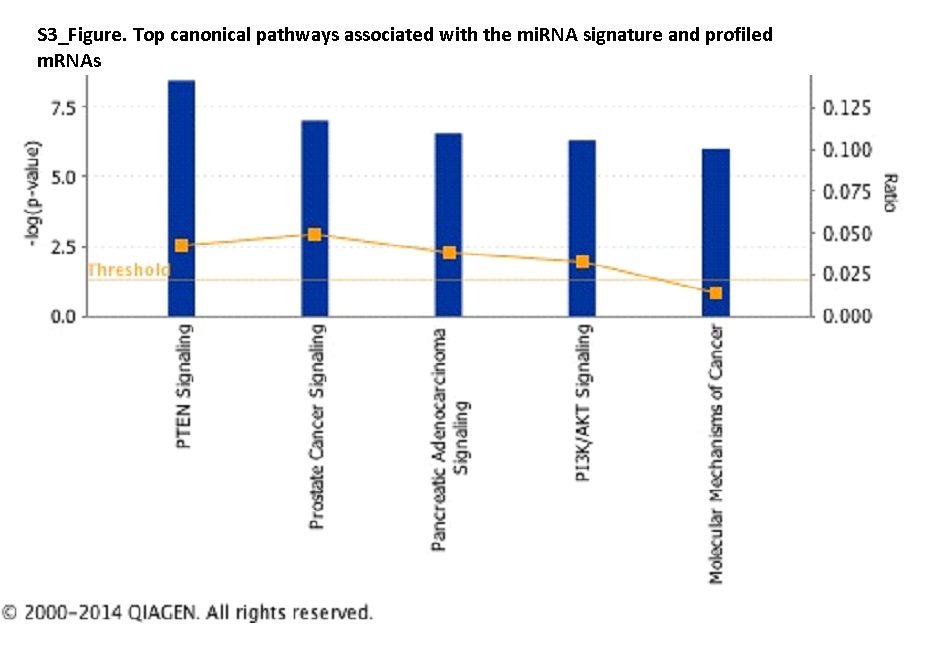

Supplement: S3 Fig — (TIFF) [file pone.0122375.s003.tiff]
