# Supplementary material for: MicroRNA Signature Characterizes Primary Tumors That Metastasize in an Esophageal Adenocarcinoma Rat Model
Source: PLoS One. 2015 Mar 31;10(3):e0122375. doi: 10.1371/journal.pone.0122375 (PMC4380408; doi:10.1371/journal.pone.0122375)
Supplement: S1 Table — (PDF) [file pone.0122375.s004.pdf]

S5_Table . Fold change in gene expression and p-value of the 4 miRNA signature and downstream/upstream targets

Fold Change	p-value	ID	Symbol	Location	Type(s)
-14.505	1.00E-04	rno-miR-32-5p	miR-92a-3p (and other miRNAs w/seed AUUGCAC)	Cytoplasm	mature microRNA
-12.584	1.81E-02	rno-miR-451-5p	miR-451a (and other miRNAs w/seed AACCGUU)	Cytoplasm	mature microRNA
-9.394	2.24E-03	rno-miR-141-3p	miR-141-3p (and other miRNAs w/seed AACACUG)	Cytoplasm	mature microRNA
-9.33	3.05E-02	rno-miR-133b-3p	miR-133a-3p (and other miRNAs w/seed UUGGUCC)	Cytoplasm	mature microRNA
1.444	1.10E-01	BCL2	BCL2	Cytoplasm	transporter
1.611	1.30E-02	KRAS	KRAS	Cytoplasm	enzyme
1.767	5.20E-03	CDKN1B	CDKN1B	Nucleus	kinase
1.824	2.62E-04	AGO2	AGO2	Cytoplasm	translation regulator
2	3.94E-02	BCL2L11	BCL2L11	Cytoplasm	other
2.013	2.61E-04	AKT1	AKT1	Cytoplasm	kinase
2.177	1.08E-02	ZEB2	ZEB2	Nucleus	transcription regulator
