# Supplementary material for: MicroRNA Signature Characterizes Primary Tumors That Metastasize in an Esophageal Adenocarcinoma Rat Model
Source: PLoS One. 2015 Mar 31;10(3):e0122375. doi: 10.1371/journal.pone.0122375 (PMC4380408; doi:10.1371/journal.pone.0122375)
Supplement: S2 Table — (PDF) [file pone.0122375.s005.pdf]

S4_Table. Top 5 canonical pathways for the the 4 miRNA signature and associated downstream/upstream molecules.

Canonical Pathway	-log(p-value)	p-value	Molecules Associated
**PTEN signaling**	8.439	3.64E-09	AKT1, BCL2, BCL2L11, CDKN1B, KRAS
**Prostate Cancer Signaling**	6.998	1E-07	AKT1, BCL2, CDKN1B, KRAS
**Pancreatic Adenocarcinoma Signaling**	6.548	2.83E-07	AKT1, BCL2, CDKN1B, KRAS
**PI3K/AKT Signaling**	6.288	5.15E-07	AKT1, BCL2, CDKN1B, KRAS
**Molecular Mechanisms of Cancer**	5.989	1.02E-06	AKT1, BCL2, BCL2L11, CDKN1B, KRAS
